# Supplementary material for: Bosonic spinons in anisotropic triangular antiferromagnets
Source: Nat Commun. 2021 Nov 9;12:6453. doi: 10.1038/s41467-021-26716-8 (PMC8578630; doi:10.1038/s41467-021-26716-8)
Supplement: Supplementary file 1 — Supplementary Information [file 41467_2021_26716_MOESM1_ESM.pdf]

Supplementary Information for

Bosonic spinons in anisotropic triangular antiferromagnets

Youngsu Choi,[1,2,4] Suheon Lee,[1,4] Je-Ho Lee,[1,4] Seungyeol Lee,[1] Maeng-Je Seong,[1,*] and Kwang-Yong Choi[1,3,*]

[1]*Department of Physics, Chung-Ang University, Seoul 06947, Republic of Korea*

[2]*Department of Energy Science, Sungkyunkwan University, Suwon 16419, Republic of Korea*

[3]*Department of Physics, Sungkyunkwan University, Suwon 16419, Republic of Korea*

[4] These authors contribute equally: Youngsu Choi, Suheon Lee, Je-Ho Lee

[*] Email to mseong@cau.ac.kr and choisky99@skku.edu

Supplementary Note 1: Temperature dependence of the phonon parameters

In Supplementary Fig. 1a, we present the Raman susceptibility $\chi''(\omega)$ in (*bb*), (*cc*), and (*bc*) polarizations measured at T = 300 and 4.3 K. The Raman susceptibility $\chi''(\omega)$ is related to the Bose-corrected Raman intensity through the relation $I(\omega) = (1 + n(\omega))\chi''(\omega)$. Here, $n(\omega)$ is a Bose function.

Before proceeding, we analyze the observed phonon modes by the factor-group theory. $Ca_3ReO_5Cl_2$ possesses the space group *Pnma* (No.62) [1]. According to the point group representation of D_{2h} (*mmm*), the factor-group analysis predicts a total of 66 Raman-active modes: $\Gamma = 20A_g(aa,bb,cc) + 13B_{1g}(ab) + 20B_{2g}(ac) + 13B_{3g}(bc)$. In the chosen polarizations $20A_g(bb, cc)$ and $13B_{3g}(bc)$ modes are symmetry-allowed. We observe a total of $22A_g$ and $8B_{3g}$ modes. Some weak peaks cannot be unambiguously assigned. We ascribe the extra modes to polarizer leakage. The missing modes may be due to the weakness of their scattering intensity or the overlap of their frequencies within a spectral resolution.

Shown in Supplementary Fig. 1b is the temperature dependence of the frequency, full width at half maximum (FWHM), and normalized intensity of the representative phonons at 518.5 cm^{-1}, 403.9 cm^{-1}, and 24.7 cm^{-1}. With decreasing temperature, the high-energy phonon modes at 518.5 cm^{-1} and 403.9 cm^{-1} exhibit a hardening by ~ 4 cm^{-1}, being consistent with lattice anharmonicity. In sharp contrast, the low-energy 24.7 cm^{-1} mode shows a small softening by ~ 1 cm^{-1} with decreasing temperature. This anomaly points to a strong coupling of the low-energy phonons to magnetic degrees of freedom.

For quantitative analysis of the observed phonon anomalies, we fit the experimental data to an anharmonic phonon interaction model [2]:

$$\omega(T) = \omega_0 + \mathrm{A}\left[1 + 2/\left(e^{\hbar\omega_0/2k_BT} - 1\right)\right], \quad (1)$$

and

$$\Gamma(T) = \Gamma_0 + \mathrm{B}\left[1 + 2/\left(e^{\hbar\omega_0/2k_BT} - 1\right)\right]. \quad (2)$$

Here, ω_0 and Γ_0 are the phonon frequency and linewidth at T=0 K, respectively, and A and B are constants. In the temperature range of T=80 – 300 K, the phonon harmonicity gives a nice description to the data with the fitting parameters A=-4.24 cm^{-1} and B=7.14 cm^{-1} for 518.45 cm^{-1} and A=-2.51 cm^{-1} and B=6.5 cm^{-1} for 403.87 cm^{-1}, and A=0.04 cm^{-1} and B=0.07 cm^{-1} for 24.7 cm^{-1}. The high-energy modes show small deviations from the fitted curves for temperatures below T = 80 K. This discrepancy becomes apparent for the low-energy 24.7 cm^{-}

[1] mode in its frequency and FWHM. As the 24.7 cm^{-1} mode lies on top of a magnetic continuum, the phonon anomaly suggests the existence of another relaxation channel in addition to an anharmonic phonon process. As discussed below, the magnetic specific heat and entropy become appreciable for temperatures below T=80 K, indicative of the development of short-range magnetic correlations. As such, a spin-phonon coupling is responsible for the renormalization of the phonon energy and lifetime.

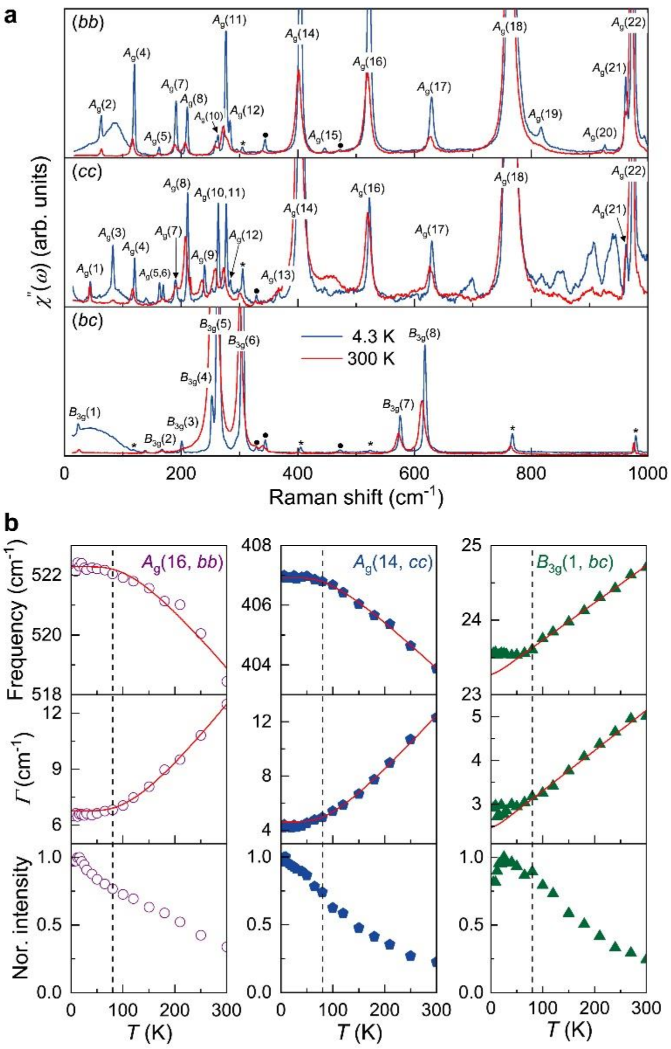

Supplementary Fig. 1 Temperature dependence of the phonon parameters of $Ca_3ReO_5Cl_2$. **a** Raman susceptibility of $Ca_3ReO_5Cl_2$ measured at T = 300 K and 4.3 K in (*bb*), (*cc*), and (*bc*) polarizations. The asteroid and dot symbols indicate phonon modes induced by polarizer leakage. **b** Temperature dependence of the frequency, the FWHM, and the normalized intensity of the 518.45 cm^{-1} ($A_g(16)$), 403.87 cm^{-1} ($A_g(14)$), and 24.7cm^{-1} ($B_{3g}(1)$) modes. The red solid lines are fits to an anharmonic phonon model. The dashed vertical line marks a dimensional crossover at T=80 K.

Supplementary Note 2: Angular dependence of phonon modes

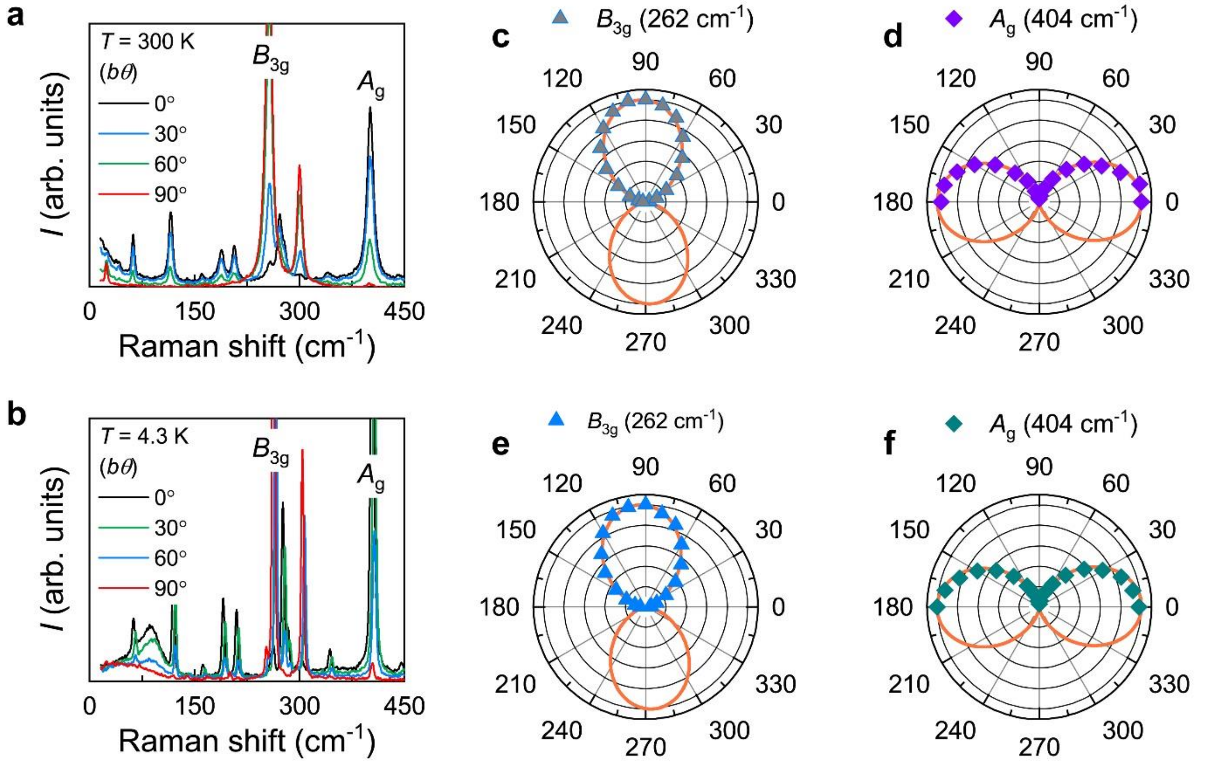

Supplementary Fig. 2 Angular dependence of phonons in (*bθ*) polarizations. **a, b** As-measured Raman spectra in cross (*bθ*) scattering configuration at *T*=300 K and 4.3 K. **c-f** Angular dependence of the normalized Raman intensity for the representative B_{3g}(262 cm^{-1}) mode and A_g (404 cm^{-1}) mode measured at *T*=300 K and 4.3 K. The orange solid curves are fits to the data as described in the text.

To figure out the symmetry of phonon excitations, we recorded the angle dependence of polarized Raman spectra for two distinct scattering geometries. In the cross (*bθ*) scattering configuration, the incident light polarization is fixed to the *b*-axis, while the scattered light polarization *θ* is rotated towards the *c*-axis with respect to the *b*-axis (intrachain direction). In the (*θθ*) configuration, the polarizer (incident light polarization) and analyzer (scattered light polarization) are simultaneously rotated in 10º increments from *θ*=0º (the *b*-axis) to 180º. Supplementary Fig. 2 and Fig. 3 summarize the angular dependence of the polarized Raman spectra of $Ca_3ReO_5Cl_2$.

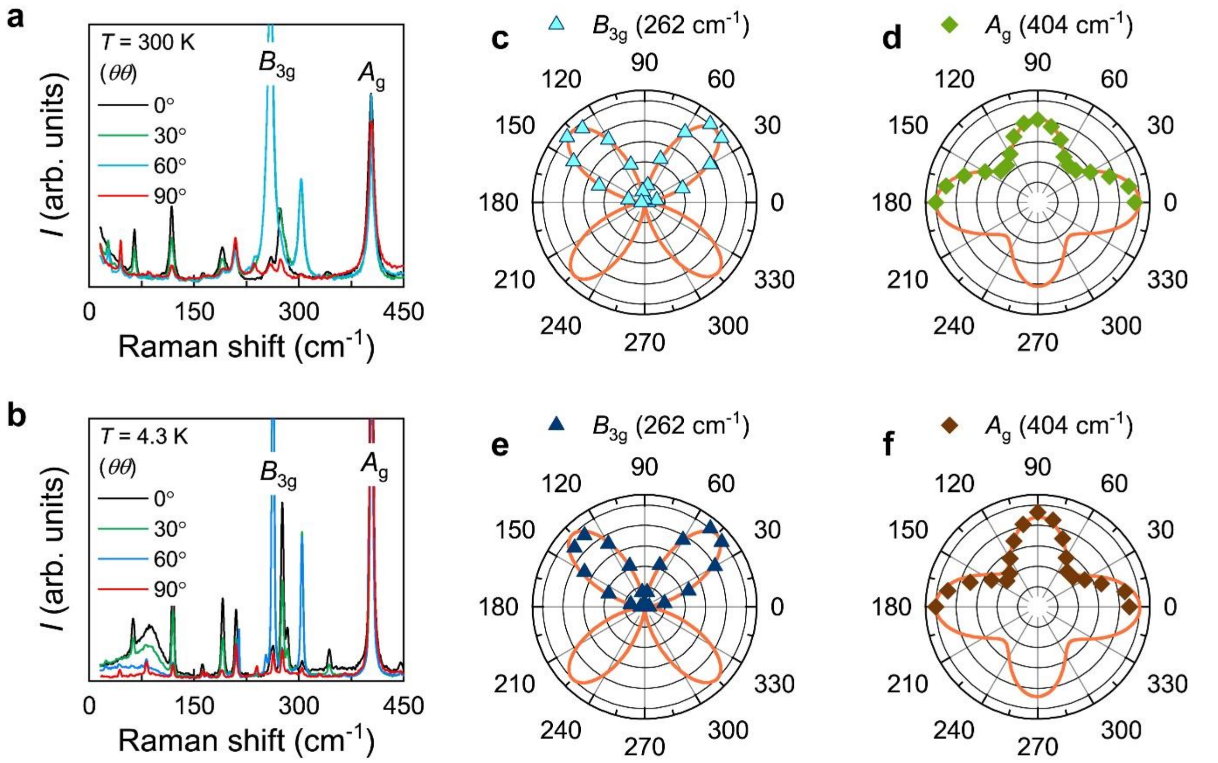

Supplementary Fig. 3 Angular dependence of Raman spectra in parallel (*θθ*) polarizations. a, b As-measured Raman spectra in parallel (*θθ*) polarization at T=300 K and 4.3 K. **c- f** Polar plots of the scattering intensities of the representative B_{3g}(262 cm^{-1}) mode and A_g (404 cm^{-1}) mode measured at T=300 K and 4.3 K. The orange solid curves are the fitting of the experimental data described in the text.

According to the aforementioned factor group analysis, we can observe the A_g and B_{3g} modes in the (*bc*) scattering geometry. Within the semiclassical theory, the Raman intensity of the phonon scatterings is given as [3]

$$I \propto |e_{\text{in}} \cdot R \cdot e_{\text{out}}|^2, \tag{3}$$

where e_{in} and e_{out} are the polarization vectors of the incident and scattered light, respectively, and R is the Raman tensor. In order to describe the angular dependence of Raman intensities, we take a light absorption effect into account [3, 4]. In an absorptive material, thus, the Raman tensors of the A_g and B_{3g} modes should be in a complex form, given as

$$R(A_g) = \begin{pmatrix} |a|e^{i\phi_a} & 0 & 0 \\ 0 & |b|e^{i\phi_b} & 0 \\ 0 & 0 & |c|e^{i\phi_c} \end{pmatrix}, \tag{4}$$

and $$R(B_{3g}) = \begin{pmatrix} 0 & 0 & 0 \\ 0 & 0 & |f|e^{i\phi_f} \\ 0 & |f|e^{i\phi_f} & 0 \end{pmatrix}. \tag{5}$$

Given that the incident and scattered light lies in the *bc* plane, the polarization unit vectors are expressed as $e_{\mathrm{in}} = (0, \ 1, \ 0)$ and $e_{\mathrm{out}} = (0, \ \cos\theta, \ \sin\theta)$ for the (*bθ*) configuration, where the angle *θ* is measured relative to the *b*-axis. Then, we obtain the angular dependence of the Raman intensity for the A_g and B_{3g} modes:

$$I(A_g) = |b|^2\cos^2\theta, \tag{6}$$

and $$I(B_{3g}) = |f|^2\sin^2\theta. \tag{7}$$

In the (*bθ*) polarization, the angular-dependent intensity of both the A_g and B_{3g} modes reveals 180° periodicity. The A_g mode vanishes when the incident and scattered polarization are perpendicular to each other. On the contrary, the intensity of the B_{3g} mode reaches a maximum in this perpendicular polarization configuration.

For the parallel polarizations, we have $e_{\mathrm{in}} = e_{\mathrm{out}} = (0, \ \cos\theta, \ \sin\theta)$. The Raman intensity for each mode is given by

$$I(A_g) = (|b|\cos^2\theta\cos\phi_{cb} + |c|\sin^2\theta)^2 + |b|^2\cos^4\theta\sin^2\phi_{cb}, \tag{8}$$

and $$I(B_{3g}) = |f|^2\sin^2 2\theta, \tag{9}$$

where $\phi_{cb} = \phi_c - \phi_b$ is the phase difference between the *b* and *c* components of the Raman tensor. From Equations (8) and (9), it is apparent that the angular dependence of the A_g and B_{3g} modes has commonly a 90° period.

We next compare the calculated intensities to the experimental angular-dependent data. Supplementary Fig. 2 and Fig. 3 show polar plots of the angular dependence of the two selected phonon modes at 262 cm^{-1} (B_{3g} mode) and 404 cm^{-1} (A_g mode). The (*bθ*) polarization data are in good agreement with the theoretical curves. The symmetry of the B_{3g} and A_g modes assigned by the factor-group analysis matches nicely with the angular variation of their intensity. In the (*bθ*) configuration, the angular dependence for the B_{3g} and A_g intensity shows no variation between T = 300 and 4.3 K, ruling out the possibility of structural transition. For the A_g mode, the calculated curves reproduce the experimentally observed angular dependence of the phonon intensity with $|c/b| = 0.90$ and the phase difference ϕ_{cb}= 90° (see the orange solid curves in Supplementary Fig. 2).

Supplementary Note 3: Angular dependence of spinon and triplon excitations

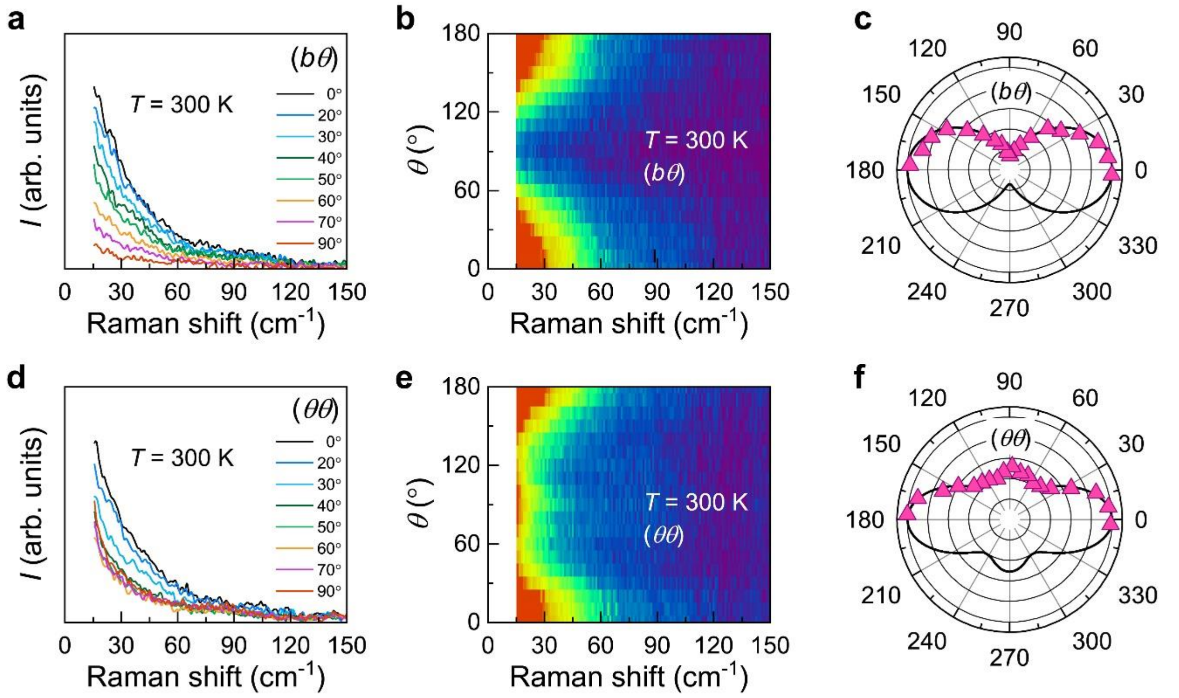

Supplementary Fig. 4 Angular dependence of Raman response in (*bθ*) and (*θθ*) configurations at *T*=300 K. **a**, **d** Angle variation of quasielastic scatterings measured at *T*=300 K in (*bθ*) and (*θθ*) polarization configurations. **b**, **e** Color plots of the magnetic Raman scattering intensity in the angle-Raman-shift plane in (*bθ*) and (*θθ*) polarizations. **c**, **f** Polar plots of the integrated magnetic continuum. The solids lines are the fitted curves described in Methods.

In Supplementary Fig. 4a,d, we present the angular dependence of phonon-subtracted Raman spectra in (*bθ*) and (*θθ*) configurations at *T*=300 K. The Raman scattering intensity is maximum at θ=0° (in the intrachain polarization). The intensity decreases continuously with increasing the angle θ=0° to 90° in two configurations. We further visualize an angular dependence of the Raman response in a color plot in Supplementary Fig. 4b,e. To analyze the symmetry of the Raman response at *T*=300K, we depict the integrated intensity in polar plots as shown in Supplementary Fig. 4c,f. The Raman responses have a 180° periodicity and are in excellent agreement with the theoretical curves (see Method in the main text).

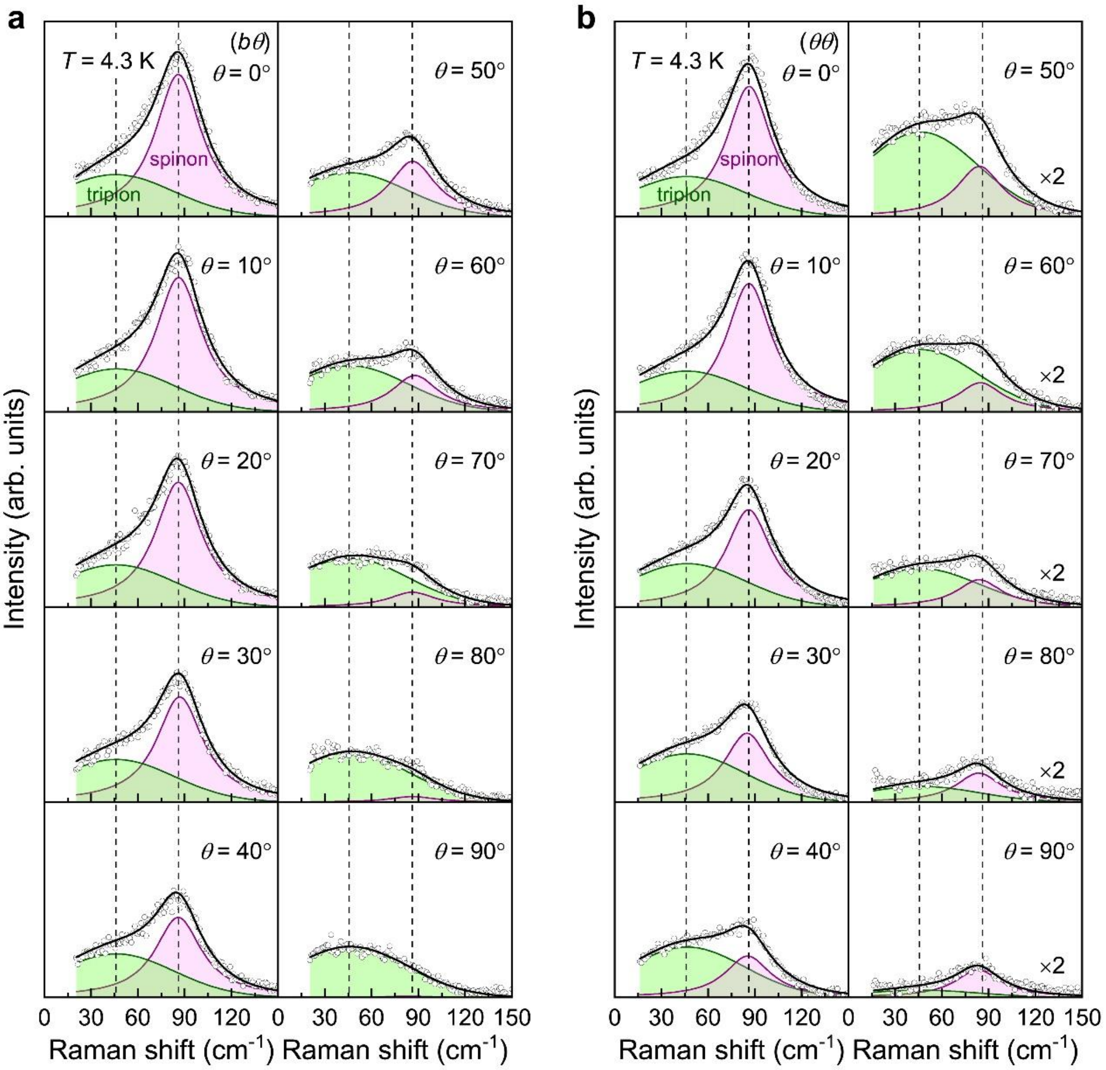

Supplementary Fig. 5 Angular dependence of spinon and triplon excitations in ($b\theta$) and ($\theta\theta$) configurations at T= 4.3 K. a, **b** Angle variation of spinon (pink shading) and triplon (green shading) components measured at T=4.3 K in ($b\theta$) and ($\theta\theta$) polarizations. We note that the spectral shape and peak energy of the spinon and triplon are independent of angle.

To analyze the angular dependence of spinons and triplons, we decompose the phonon-subtracted Raman spectra at T=4.3 K into two components (denoted by S and T in Fig. 1e,f of the main text and Supplementary Fig. 5). The T component in (bc) polarization originates mainly from triplons. The S component involves largely two-pair spinon-antispinon excitations. First, we fit the T component to a Gaussian profile, yielding the peak frequency $\omega \sim 46$ cm^{-1}

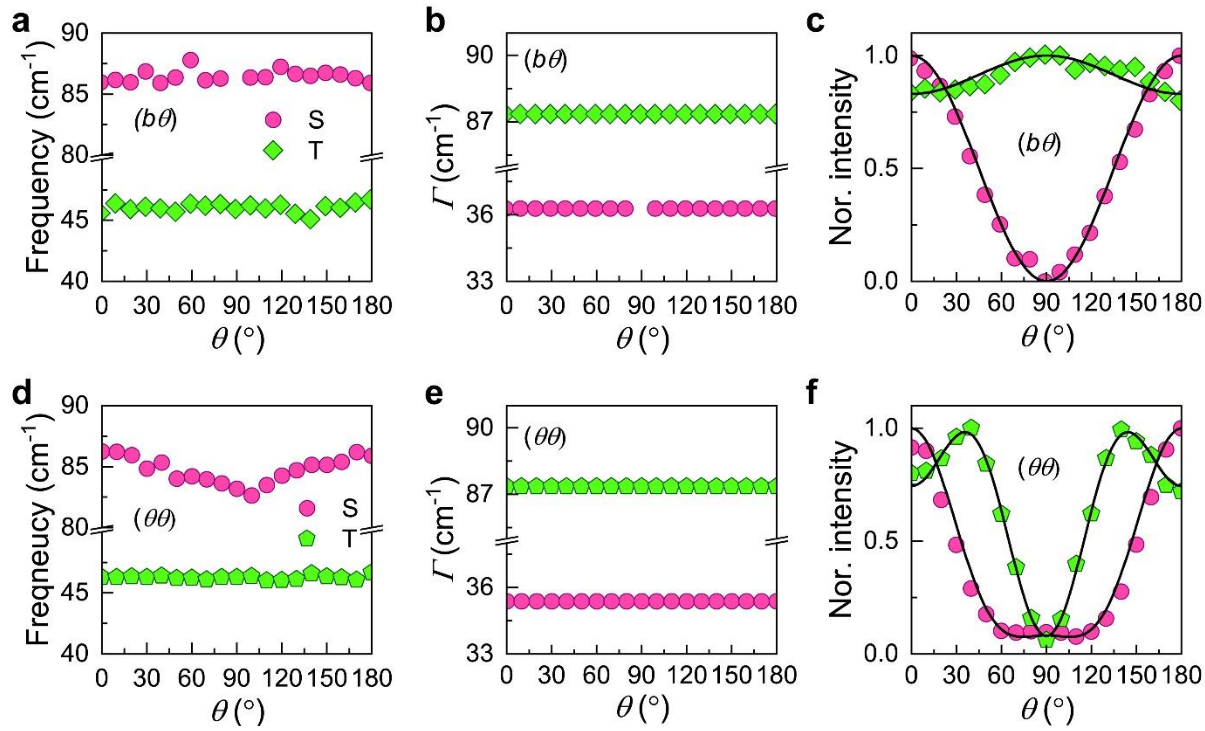

Supplementary Fig. 6 Angular dependence of spinon and triplon excitations. Angular dependence of the frequency (**a**, **d**), the full width at half maximum (**b, e**), and the normalized intensity (**c**, **f**) of the spinon (S) and triplon (T) excitations in ($b\theta$) and ($\theta\theta$) polarizations. The solid lines represent the angular dependence fitting.

and FWHM Γ~87 cm^{-1}. Next, the Raman spectrum in (*bb*) polarization is described by a sum of Lorentzian and Gaussian profiles. In doing that, we fix the T component using the fitting parameters obtained from the (*bc*) polarization data.

Supplementary Fig. 6 exhibits an angle dependence of the frequencies, FWHMs, and normalized intensities of the S and T modes in ($b\theta$) and ($\theta\theta$) configurations. In ($b\theta$) polarization, the frequencies and FWHMs of the S and T excitations are independent of θ, guaranteeing the validity of our decomposition model. In contrast, the normalized intensities show a notable angle variation. The S excitation has the A_1 symmetry and the angle dependence of its intensity follows $I_{\mathrm{S}}(\theta) \sim \cos^2\theta$ (see Methods in the main text). On the other hand, the T excitation features the A_1+A_2 symmetry, following $I_{\mathrm{T}}(\theta) = |r_2|^2\cos^2\theta + |r_4|^2 \sin^2\theta$ with $|r_4/r_2| =$ 1.09. In ($\theta\theta$) polarization, the frequency of the T excitation shows little change with θ while the frequency of the S excitation slightly decreases with increasing angle toward θ=90°, the interchain (*cc*) polarization. In the same manner as ($b\theta$) polarization, the angle-dependent

intensity of the S excitation is described by the A_1 symmetry with $|r_3/r_2| = 0.28$. The intensity of the T excitation is represented by A_1+A_2 symmetry whose angle variation is given by $|r_3/r_2| = 0.32$ and $|r_4/r_2| = 0.98$.

Supplementary Note 4: Temperature dependence of the dynamic Raman susceptibility

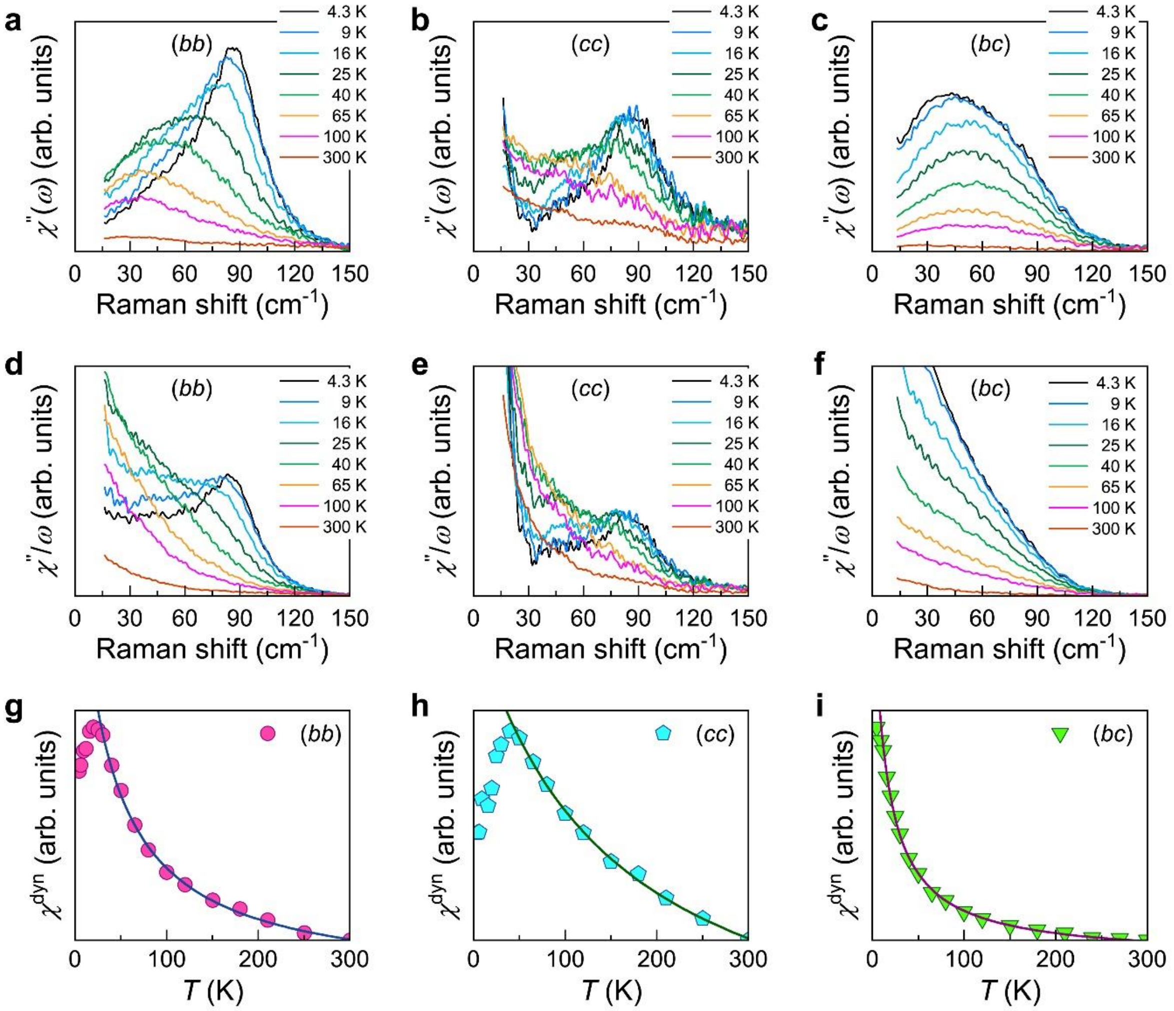

Supplementary Fig. 7 Temperature dependence of the dynamic Raman susceptibility. a-c Temperature dependence of the Raman susceptibility $\chi''(\omega)$ in (*bb*), (*cc*), and (*bc*) polarizations. **d-f** Temperature dependence of the Raman conductivity $\chi''(T)/\omega$. **g-i** Temperature dependence of the dynamic Raman susceptibility derived from $\chi''(T)/\omega$. The solid lines are fits to the Curie-Weiss law.

In Supplementary Fig. 7a-c, we present Raman susceptibility $\chi''(\omega)$ versus temperature in (*bb*), (*cc*), and (*bc*) polarizations, focusing on the low-energy magnetic excitations. We further

plot the temperature dependence of the Raman conductivity χ''/ω in Supplementary Fig. 7d-f. The Raman conductivity features a pronounced peak centered at ω=0 cm^{-1} in a high-T hydrodynamics regime. $\chi''(T)/\omega$ exhibits a strong enhancement with decreasing temperature in (bc) polarization. On the other hand, $\chi''(T)/\omega$ in (bb) and (cc) polarization exhibits a maximum at 86 cm^{-1} below T~25 K. From the Raman conductivity, we can drive a dynamic Raman susceptibility using Kramers-Kronig relation $\chi^{\text{dyn}} = \lim_{\omega\to 0}\chi(\mathbf{k}=0,\omega) \equiv \frac{2}{\pi}\int_0^\infty \frac{\chi''(\omega)}{\omega}d\omega$ by integrating up to 150 cm^{-1}. The dynamic Raman susceptibility is empirically approximated by the Curie-Weiss law, $\chi^{\text{dyn}}(T) \propto 1/(T-\Theta)$ in Supplementary Fig. 7g-i. $\chi^{\text{dyn}}(T)$ shows a broad maximum at T=25 K for (bb) and T=50 K for (cc), heralding the crossover to a Tomonaga-Luttinger liquid state. In contrast, the (bc) $\chi^{\text{dyn}}(T)$ for the triplon channel diverges as $T\rightarrow$ 0 K, implying a quantum paramagnetic behavior down to zero temperature.

Supplementary Note 5: Critical spin dynamics and magnetic specific heat

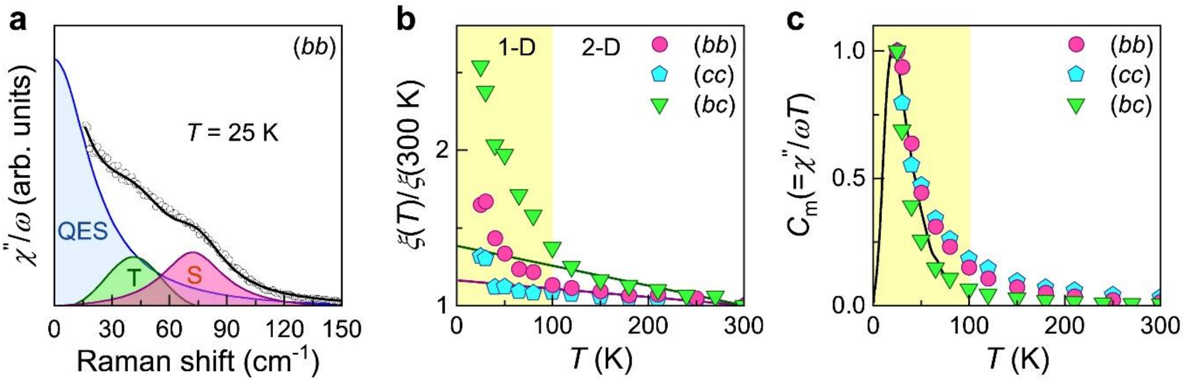

Supplementary Fig. 8 Quasielastic response, spin correlation length, and magnetic specific heat. a Representative Raman conductivity $\chi''(\omega)/\omega$ in (bb) polarization at T=25 K, which is composed of quasielastic scattering (QES), spinon (S), and triplon (T) components. **b** Temperature dependence of the spin-correlation length extracted from the Raman conductivity in (bb), (cc), and (bc) polarizations. The solid lines are linear fits to the high-temperature data. **c** Magnetic specific heat C_m evaluated from the relation $C_m(T) \propto \chi''(\omega)/(\omega T)$. The solid line is the thermodynamic magnetic specific heat taken from Ref. [1].

In a quasi-1D spin system, quasielastic light scattering arises from diffusive magnetic fluctuations [5-7] or fluctuations of the spin energy density [8,9]. We find that our quasielastic response is described by a Lorentzian spectral function, implying that the fluctuations of the

magnetic energy density provide a dominant contribution. In this case, we can calculate the scattering intensity using the Fourier components of a correlation function of the magnetic energy density

$$I(\omega) \propto \int_{-\infty}^{\infty} e^{-i\omega t} dt \langle E(k,t) E^*(k,0) \rangle. \tag{10}$$

In the high-temperature and hydrodynamical conditions [8, 10], Equation (10) is simplified to

$$I(\omega) \propto \frac{C_m T^2 D_T k^2}{\omega^2 + (D_T k^2)^2}, \tag{11}$$

where C_m is the magnetic specific heat, D_T is the thermal diffusion coefficient $D_T = K/C_m$, and K is the magnetic thermal conductivity. Equation (11) can be rewritten in terms of a Raman susceptibility $\chi''(\omega)$

$$\frac{\chi''(\omega)}{\omega} \propto \frac{C_m T D_T k^2}{\omega^2 + (D_T k^2)^2}. \tag{12}$$

In this light, the magnetic specific heat can be extracted from the Raman conductivity $\chi''(\omega)/\omega$. In Supplementary Fig. 7 and Fig. 8a, we plot the temperature dependence of $\chi''(\omega)/\omega$, marked with the quasielastic component (blue shading), in (*bb*), (*cc*), and (*bc*) polarizations.

Supplementary Fig. 8b, c shows the temperature dependence of the spin correlation length ξ and the magnetic specific heat C_m. $\xi(T)$ is obtained by the inverse of the FWHM of the Lorentzian profile. $C_\mathrm{m}(T)$ is evaluated from the relation $C_m(T) \propto \chi''(\omega)/(\omega T)$. On cooling down to 80 K, $\xi(T)$ displays a weak linear *T*-increase and then shows a rapid increase below 80 K, which is common to the three polarizations. Basically, the spin correlation length in the paramagnetic phase is temperature-independent. Thus, this is associated with a dimensional crossover from 1D and 2D, as inferred from the static magnetic susceptibility in Supplementary note 1. A close comparison reveals that for temperatures below 80 K, $\xi(T)$ is more pronounced in (*bc*) polarization than in (*bb*) and (*cc*) polarizations. This reflects that the nature of quasiparticle excitations differs between the parallel and cross polarizations.

Next, we compare the spectroscopically derived $C_\mathrm{m}(T)$ with the thermodynamic magnetic specific heat (the solid line taken from Ref. [1]). The spectroscopic and thermodynamic data are comparable for temperatures below 80 K. Unlike the thermodynamic specific heat, however, the spectroscopically derived C_m has an appreciable contribution in the paramagnetic range of

T>100 K, especially in parallel (*bb*) and (*cc*) polarizations. This suggests that quasi-2D diffusive fluctuations contribute additionally to the quasielastic scattering in the high-T paramagnetic regime.

Supplementary Note 6: Magnetic susceptibility

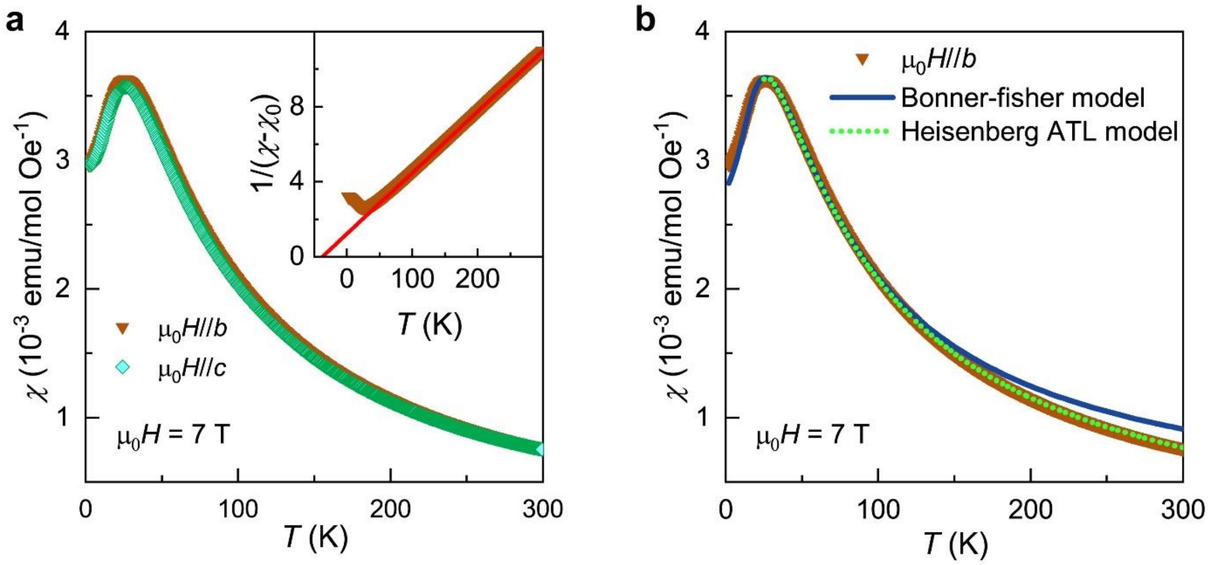

Supplementary Fig. 9 Temperature dependence of magnetic susceptibility. a Temperature dependence of the magnetic susceptibility $\chi(T)$ of single-crystal $Ca_3ReO_5Cl_2$ measured under a magnetic field of μ_0H=7 T for $\mu_0H//b$ and $\mu_0H//c$. The inset shows the inverse magnetic susceptibility of $1/(\chi- \chi_0)$ along the *b*-axis. Here, χ_0 is the temperature-independent term. The solid red line is a Curie-Weiss fit. **b** Theoretical analysis of $\chi(T)$ for $\mu_0H//b$ in terms of the Bonner-fisher model (solid blue line) and the Heisenberg ATL model (dotted green line). The fitting parameters are described in the text.

Supplementary Fig. 9 shows the temperature dependence of the *dc* magnetic susceptibility $\chi(T)$ of $Ca_3ReO_5Cl_2$ for $\mu_0H//b$ and $\mu_0H//c$. We measured $\chi(T)$ in an external magnetic field of μ_0H=7 T. For both field directions, $\chi(T)$ exhibits a broad maximum at T_{max}=27 K, which is typical for the short-range ordering in low-dimensional spin systems. We observe no discernible anomaly pertinent to long-range magnetic order down to 2 K. Despite the apparent anisotropic magnetic sublattice, the in-plane magnetic anisotropy is not bigger than 3 %. This suggests that the Re^{6+} spins are predominantly exchange-coupled by Heisenberg-type interactions.

In the high-temperature range of T=150 – 300 K, the Curie-Weiss fits yield the Curie constant C=0.30707 emu·mol^{-1}·Oe^{-1}, the Curie-Weiss temperature Θ_{CW}=-37.3 K, and the T-independent term χ_0=-1.50197×10^{-4} emu·mol^{-1}·Oe^{-1} for $\mu_0 H//b$ and C=0.28724 emu·mol^{-1}·Oe^{-1}, Θ_{CW}=-32.2 K, and χ_0=-1.16332×10^{-4} emu·mol^{-1}·Oe^{-1} for $\mu_0 H//c$ [see the inset of Supplementary Fig. 9a]. The negative Curie-Weiss temperature indicates predominant antiferromagnetic exchange interactions between the Re^{6+} spins. The effective magnetic moments are evaluated to be μ_{eff}=1.567 μ_B for $\mu_0 H//b$ and μ_{eff}=1.515 μ_B for $\mu_0 H//c$. These values are rather smaller than the spin-only value of 1.73 μ_B for S=1/2, indicating the presence of substantial spin-orbit coupling. All the obtained parameters are in perfect agreement with the previous result [11].

To examine the one-dimensionalization induced by geometrical frustration, we attempted to fit $\chi(T)$ using two different models: a S=1/2 Heisenberg anisotropic triangular lattice (ATL) model for high T versus a Bonner-Fisher model for low T [12,13]. In the temperature range of T=25–300 K, $\chi(T)$ for $\mu_0 H//b$ is well reproduced by the Heisenberg ATL model with J= 38.48 K and J'=13.6 K, (J/J'=0.35) with g=1.81. However, this model provides a poor description of $\chi(T)$ below 25 K. On the other hand, the Bonner-Fisher model with the magnetic parameters J=40.6 K and g=1.60 well describes $\chi(T)$ below 100 K, but not for above 100 K. The disparate behaviors of $\chi(T)$ allude to the dimensional reduction from a quasi-two-dimensional ATL to a quasi-one-dimensional spin chain at about T=100 K.

Supplementary References

[1] Hirai, D. et al. "Visible" 5*d* orbital states in a pleochroic oxychloride. *J. Am. Chem. Soc.* **31**, 139 (2017).

[2] Balkanski, M., Wallis, R. F., & Haro, E. Anharmonic effects in light scattering due to optical phonons in silicon. *Phys. Rev. B* **28**, 1928 (1983).

[3] Loudon, R. The Raman effect in crystals. *Advances in Physics* **13**, 423-482 (1964).

[4] Cardona, M. In Light scattering in Solids II. Vol. 50, Ch. 2, 19-278, *Springer* (1982).

[5] Rechards, P. M., & Brya, W. J. Spin-fluctuation light scattering at high temperature. *Phys. Rev. B* **9**, 3044 (1974).

[6] van Loosdrecht et al. A. Inelastic Light Scattering from Magnetic Fluctuations in $CuGeO_3$. *Phys. Rev. Lett.* **76**, 311 (1996).

[7] Narozhny, B. N. Spin diffusion in one-dimensional antiferromagnets. *Phys. Rev. B* **54**, 3311 (1996).

[8] Halley, J. W. Light Scattering as a Probe of Dynamical Critical Properties of Antiferromagnets. *Phys. Rev. Lett.* **41**, 1605 (1978).

[9] Reiter, G. F. Light scattering from energy fluctuations in magnetic insulators. *Phys. Rev. B* **13**, 169 (1976).

[10] Halperin, B. I., & Hohenberg, P. C. Hydrodynamic Theory of Spin Waves. *Phys. Rev*. **188**, 898 (1969).

[11] Hirai, D., Nawa, K., Kawamura, M., Misawa, T., & Hiroi, Z. One-dimensionalization by Geometrical Frustration in the Anisotropic Triangular Lattice of the 5*d* Quantum Antiferromagnet $Ca_3ReO_5Cl_2$. *J. Phys. Soc. Jap.* **88**, 044708 (2019).

[12] Baker, G. A. Application of the Padé Appoximant Method to the Investigation of Some Magnetic Properties of the Ising model. *Phys. Rev.* **124**, 768 (1961).

[13] Johnston, D. C. et al. Thermodynamics of spin S=1/2 antiferromagnetic uniform and alternating-exchange Heisenberg chains. *Phys. Rev. B* **61**, 9558 (2000).
